# Sodium glucose cotransporter 2 inhibitor dapagliflozin depressed adiposity and ameliorated hepatic steatosis in high-fat diet induced obese mice

**DOI:** 10.1080/21623945.2021.1979277

**Published:** 2021-09-22

**Authors:** Tuo Han, Yajie Fan, Jie Gao, Mahreen Fatima, Yali Zhang, Yiming Ding, Liang Bai, Congxia Wang

**Affiliations:** a Department of Cardiology, The Second Affiliated Hospital of Xi’an Jiaotong University, Xi’an, Shaanxi, China; b Laboratory Animal Center, Xi’an Jiaotong University Health Science Centre, Xi’an, Shaanxi, China

**Keywords:** Dapagliflozin, high-fat diet, hepatic steatosis, adiposity, browning

## Abstract

With the increasing obesity prevalence, the rates of obesity-related diseases, including type 2 diabetes, non-alcoholic fatty liver disease (NAFLD), and cardiovascular diseases, have increased dramatically. Dapagliflozin, one of the sodium glucose cotransporter inhibitors, not only exerts hypoglycaemic effects through increasing urinary glucose excretion but alsoreprograms the metabolic system, leading to benefits in metabolic and cardiovascular diseases. In this study, pre-established obese mice on a high-fat diet were given dapagliflozin by gavage for fourweeks. It showed that dapagliflozin can enhance fat utilization and browning of adipose tissue and improve local oxidative stress, thus inhibiting fat accumulation and hepatic steatosis without disturbance in body weight or plasma glycolipid level. Overall, our study highlights the potential clinical application of SGLT2 inhibition in the prevention of obesity and related metabolic diseases, such as insulin resistance, NAFLD, and diabetes.

## 1. Introduction

With the increasing prevalence of obesity around the world, the rates of serious obesity-related diseases, including diabetes, cardiovascular diseases, and non-alcoholic fatty liver disease (NAFLD), have also increased dramatically [1–3]. Obesity is closely associated with chronic activation of inflammatory states and insulin resistance, accompanied by excessive fat accumulation of body fat [4,5]. Excessive fat accumulation increases the infiltration of pro-inflammatory immune cells into metabolic tissues and causes phenotypic shifts in macrophages, leading to the aggravation of insulin resistance [6]. However, therapeutic approaches for improving systemic energy balance and chronic inflammation in obesity are still limited.

Adipose tissue can be broadly divided into white adipose tissue(WAT) and brown adipose tissue(BAT); the former is the main site for lipid synthesis and storage, while the latter is the main site for non-shivering thermogenesis for its high expression of uncoupling protein 1(UCP-1) [7,8]. The WAT includes subcutaneous adipose tissue (sWAT), such as inguinal adipose tissue (iWAT), and intra-abdominal adipose tissue, such as gonadal adipose tissue (gWAT) [7]. Besides, a new type of brown-like adipocyte, called beige cells, is recently discovered within the WAT [9]. Beige cells can be activated upon cold exposure or adrenergic stimulation – adipose tissue browning, to utilize the lipids stored in fat cells for thermogenesis [9,10]. Thus, WAT browning is regarded as a promising strategy for obesity prevention and treatment.

Sodium glucose cotransporter-2 (SGLT2) inhibitors have emerged as a promising new class of glucose-lowering drugs for the management of type 2 diabetes [11], and dapagliflozin is the most selective, reversible, and orally active SGLT2 inhibitor for wide clinical use [12]. SGLT2 is predominantly expressed in the proximal convoluted renal tubule and is responsible for approximately 90% of glucose reabsorption by the kidney [13]. Dapagliflozin exclusively inhibits the reabsorption of glucose in kidney and increases urinary glucose excretion, thereby reducing hyperglycaemia and weight with apparent pleiotropic effects [14]. Large clinical studies have proved that dapagliflozin can significantly decrease the rates of mortality and hospitalization in heart failure patients with or without diabetes [15–17]. Those benefits seem to be attributed to the adaptive metabolic improvements involving glucose fluxes, hormonal responses, and fuel shift [18], although the specific mechanisms are still unclear.

Previous studies have researched the role of several SGLT2 inhibitors in diabetes treatment using *db/db* mice [19,20] or STZ-induced diabetic model [21,22], while few of them have explored its potency on nondiabetic obesity. Therefore, this study aimed to examine the effects of the SGLT2 inhibitor dapagliflozin on obesity and its comorbidities such as NAFLD in high-fat-diet (HFD)-induced obese (DIO) mice.

## 2. Materials and methods

### 2.1. Animals and diets

Eight-week-old male C57BL/6 J mice (23.0 ± 1.8 g) in the specific pathogen-free animal room were randomly grouped into standard chow diet (SCD) (n = 7) and high-fatdiet (HFD, 60% kcal fat; ReadyDietech) (n = 13). HFD group mice were fed with HFD for 4 weeks. Then, the mice were treated with an intragastric administration of 1 mg/kg dapagliflozin (Sigma-Aldrich; HFD-DAPA) or vehicle in 10% DMSO‐containing saline (1 mL/kg total volume; HFD-DMSO) at around 10:00 AM daily continued with 4 weeks of HFD feeding. SCD group mice were fed with chow diet. After 4 weeks, an equal dose of vehicle was administered intragastrically daily, and then, the chow diet feed was continued for 4 weeks. All mice were maintained in 12 h light/dark cycle and provided with water ad libitum. 1.5% sodium pentobarbital intraperitoneal injection was used to euthanize the mice. Liver, subcutaneous (sWAT), gonadal (gWAT), and brown adipose tissues (BAT) were dissected and divided into portions for histology or mRNA analysis. All animal procedures were performed in accordance with the guidelines of the Animal Care and Use Committee of Xi’an Jiaotong University.

### 2.2 Isolation of adipocytes and SVFs

Stromal-vascular fractions (SVFs) and mature adipocytes were isolated from gonadal adipose tissue as described [23]. In brief, gonadal adipose tissue was dissected from another batch’s 8-week-old C57BL/6 J mice and then digested with collagenase II (0.25 mg/ml, Sigma) in a buffer containing 0.123 M NaCl, 5 mM KCl, 1.3 mM CaCl_2_, 5 mM glucose, 100 mM HEPES, and 4% BSA for 1 h at 37°C. After adding DMEM medium with 10% FBS to stop digestion, the cells were filtered through a nylon mesh, and the suspension was centrifuged for 1 min at 500× *g*. A 1 mL syringe was used to carefully aspirate the lower layer of the liquid while keeping the floating adipocytes untouched. Thereafter, adipocytes were harvested. The adipocytes and the SVFs were washed 2–3 times with PBS and centrifuged for 5 min at 500× *g* to collect the SVFs for total RNA extraction.

### 2.3 Biochemical Analyses

Blood was obtained from retro-orbital veins and centrifuged for 15 min at 3000 rpm to collect the plasma for biochemical analyses. Plasma triglyceride (TG) and total cholesterol (TC) levels were measured using commercial kits (Cat: 192,061; Cat: 198,061, Biosino Bio-Technology Inc, Beijing, China), and plasma superoxide dismutase (SOD) and malondialdehyde (MDA) levels were measured using assay kits (Cat: A003-1; Nanjing Jiancheng Bioengineering Institute, China) according to manufacturer’s instructions. For the random glucose tests, blood samples were collected from the mice tail tip and tested for glucose levels by using a One Touch glucose metre (LifeScan) half an hour before dapagliflozin administration. For the fasting blood glucose, mice were fasting overnight for 14 h and the glucose levels were tested in the same way mentioned above. On the last day of the experiment, the mice were given a single dose of 1 mg/kg dapagliflozin by gavage at 8 AM and fasted for 6 h to verify its hypoglycaemic effect. The blood glucose was tested using the glucose metre.

### 2.4 Histopathological examination

Liver and adipose tissues were fixed in 4% paraformaldehyde for 48 h, embedded in paraffin, and cut into sections of 5 μm thickness. Tissue sections were stained with haematoxylin–eosin (H&E) according to the protocol. Part of each liver was also embedded in optimal cutting temperature (OCT) compound for frozen sections at 5–6 μm thickness. Frozen liver sections were then stained with an Oil Red O staining (Sigma-Aldrich) for observing the lipid deposition. The sections for microscopic quantification were photographed under a microscope equipped with a digital camera (Nikon, Tokyo, Japan) at a magnification of 10× for eyepiece and 10× or 20× for objective.

### 2.5 RNA extraction and quantitative real-time PCR (qPCR)

Total RNA of tissues or separated SVFs and adipocytes was extracted by using RNAex Pro Reagent (Accurate Biology, Changsha, China). The concentration and quality of RNA were detected by using a NanoDrop2000 spectrophotometer. Reverse transcription was performed from 500 ng of total RNA by using a Evo M-MLV RT-PCR Kit (Accurate Biology, Changsha, China). Quantitative real-time PCR (qPCR) was performed as described previously [24]. Each pair of primers in our experiment was designed by previous fellows or ourselves using NCBI primer-BLAST, with the organism as ‘mouse (taxid:10,090)’ and product size between 70 and 200 nt. For real time PCR reactions, 3 μL of cDNA was added to a total of 20 μL volume using a SYBR Green Kit (Accurate Biology, Changsha, China). β-actin was used as the reference for each gene relative expression. PCR reactions were performed by using a Thermal Cycler Dice Real Time System TP-800 (TaKaRa Biology Inc., Shiga, Japan). The generation of specific PCR products was confirmed by melting curve analysis, and gene expression levels were calculated by the comparative Ct method, X = 2^−ΔΔCt^. Primers used for real-time PCR are shown in Table S1.

### 2.6 Statistical analysis

All data were expressed as the mean ± SEM. Student’s *t*-test was used for comparing two independent groups. One-way ANOVA followed by the recommended Tukey test for multiple comparisons in GraphPad software (GraphPad PRISM 8.0) was used for multiple comparisons versus the control group. *p* < 0.05 was considered statistically significant.

## 3 Results

### 3.1 Scl5a1 *was overexpressed, while* Scl5a2 *kept constant in adiposity*

**Figure 1.**
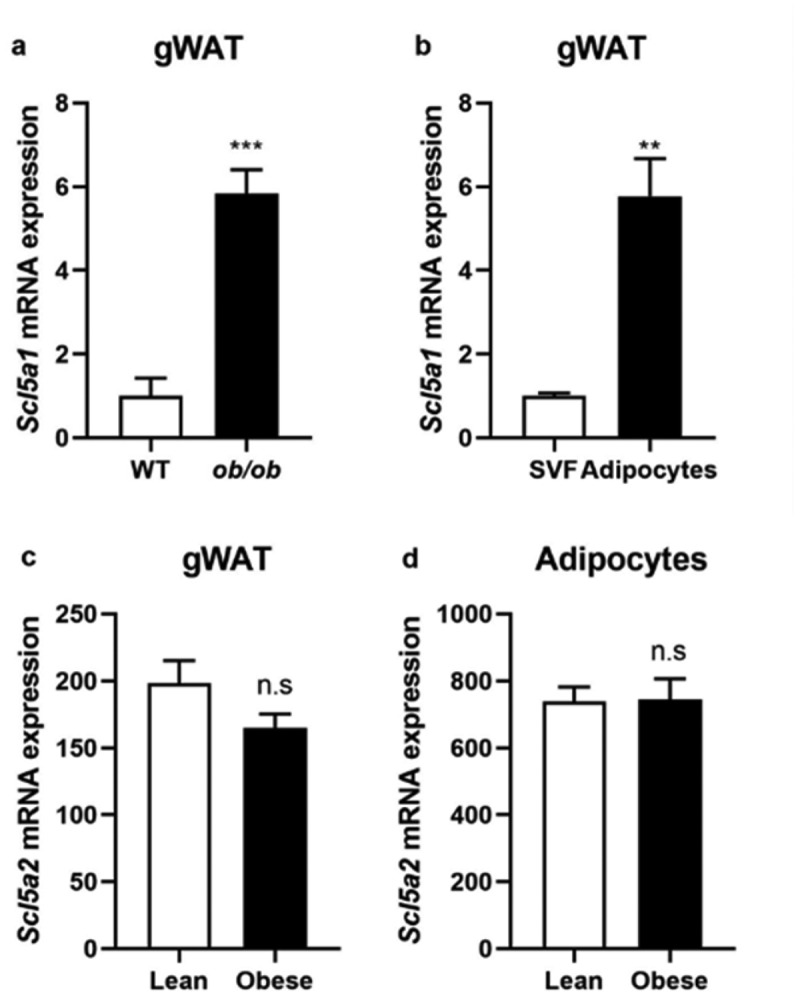
*Scl5a1* and *Scl5a2* expression in gWAT and adipocytes. (a) *Scl5a1* expression in gWAT or (b) in adipocytes and SVFs (n = 3 per group). (c) *Scl5a2* expression in the gWAT of obese rats (data adopted from GDS2946/1,368,414, n = 7–8 per group) or (d) in adipocytes from obese individuals (data adopted from GDS1497/68,635, n = 9–10 per group). Data are presented as means ± SEM. *P < 0.05, **P < 0.01, ***P < 0.001; n.s-no significant

The glucose sodium cotransporter has two subtypes, SGLT1 and SGLT2, which are encoded by *Scl5a1* and *Scl5a2* genes, respectively. SGLT1 is mainly distributed in the small intestine and distal renal tubules, while SGLT2 is mainly distributed in the proximal renal tubules [11]. However, the expression of SGLT1/2 in the adipose tissue is not yet very clear, and its association with obesity has not been reported. Therefore, we first detected the mRNA expression level of *Scl5a1* and *Scl5a2* in gonadal adipose tissue of two-month-old *ob/ob* mice, which were previously exacted and stored in our lab, and the tissues from OB/OB littermate mice were used as controls. It was found that the expression of *Scl5a1* was significantly increased compared with normal control (Figure 1a). We further isolated mature adipocytes and stromal-vascular fractions (SVFs) from the epididymal WAT of eight-week-old C57BL/6 J mice and found that *Scl5a1* was mainly expressed in adipocytes other than SVFs (Figure 1b). However, we failed to detect *Scl5a2* mRNA expression neither in WAT nor in adipocytes even using two different pairs of the primer sequence (see Table S1). Searching in the GEO database (https://www.ncbi.nlm.nih.gov/geo/), we found two sets of sequencing data: in the gWAT of rats on a high-fat-diet (GDS2946/1,368,414) and adipocytes isolated from obese individuals (GDS1497/68,635), there was no significant difference in *Scl5a2* mRNA expression between the lean control and obese groups (Figure 1c,d). Different expression patterns of SGLT1/2 in obesity may suggest their different roles in adiposity.

### 3.2 Dapagliflozin inhibited adiposity in DIO mice

**Figure 2.**
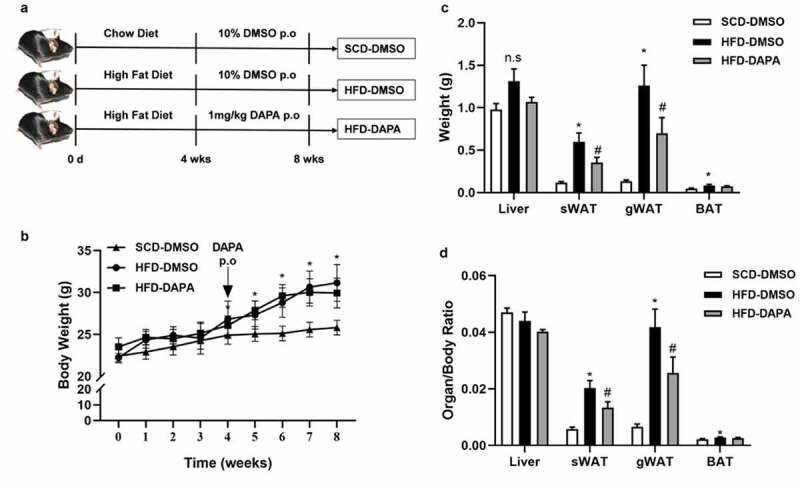
Dapagliflozin inhibited adiposity in DIO mice. (a) Scheme of the experiment design. (b) Body weights. (c) Liver and fat tissue weights. (d) Organ weight/body weight ratios. Data are presented as means ± SEM, n = 6–7 per group. *P < 0.05 HFD-DMSO vs. SCD-DMSO; ^#^*P* < 0.05 HFD-DAPA vs. HFD- DMSO at each specific set time. p.o means per os

C57BL/6 J mice were fed on a high-fat diet (HFD) or normal chow diet (SCD) for 8 weeks and were given 1 mg/kg dapagliflozin or equivalent amount of solvent as control by gavage from the 4th week (Figure 2a). The body weight of mice in the HFD group was significantly higher than that of the mice in the SCD group from the 4th week, and it turned to be more obvious in the following weeks. Dapagliflozin treatment for 4 weeks slightly inhibited the body weight gain when compared with that of the HFD-DMSO group (Figure 2b). On the organ level, there was no significant difference in liver weight among the three groups, but the weight of sWAT, gWAT, and BAT in the HFD group was significantly increased compared to the SCD group, while sWAT and gWAT weights in the HFD-DAPA group were lower than tose in the HFD-DMSO group, but still significantly higher than that in the SCD group (Figure 2c,d). These observations suggest that dapagliflozin treatment could inhibit adiposity in DIO mice.

### 3.3 Dapagliflozin decreased plasma glucose transiently and exerted no impact on plasma lipids in DIO mice

**Figure 3.**
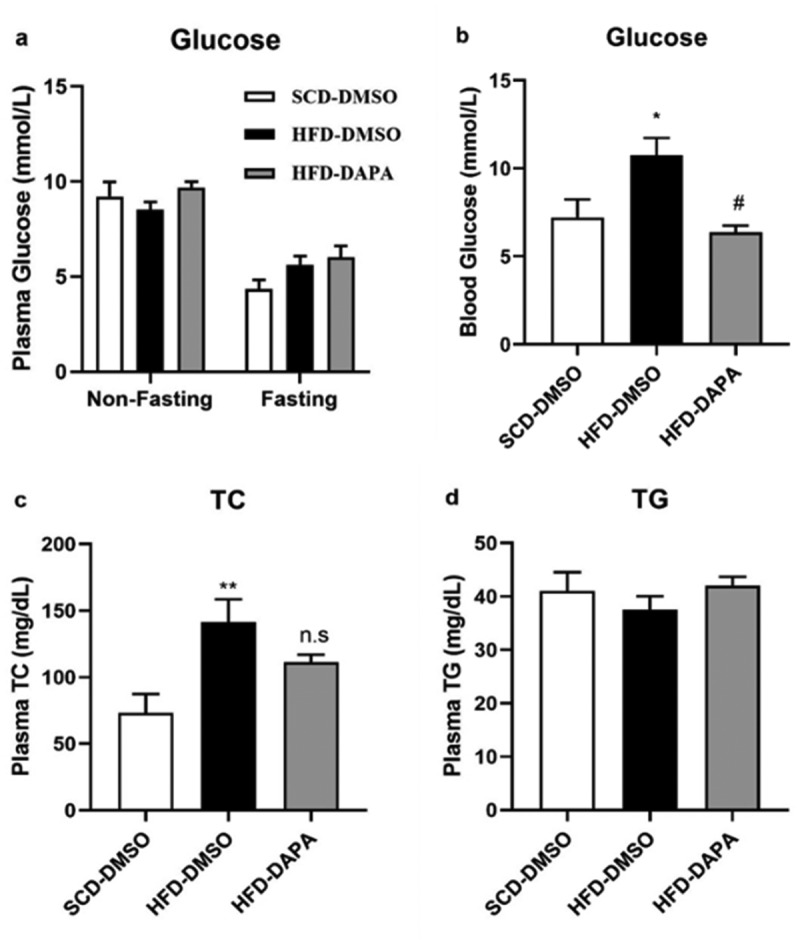
Effects of dapagliflozin on blood glucose and lipids in DIO mice. (a) Blood glucose with or without fasting. (b) Blood glucose after single gavage of dapagliflozin. (c) Plasma TC and (d) plasma TG. Data are presented as means ± SEM, n = 6–7 per group. *P < 0.05, **P < 0.01 vs. SCD-DMSO; ^#^*P* < 0.05 vs. HFD-DMSO

In order to explore the effects of dapagliflozin on glucose homoeostasis, we tested the mice blood glucose with or without fasting and found that there was no significant difference among the three groups in both conditions (Figure 3a). To verify its hypoglycaemic effect, mice were given a single dose of 1 mg/kg dapagliflozin by gavage at 8 AM on the last day and fasted for 6 h. The blood glucose concentrations were then tested. It was found that the blood glucose level in the HFD-DMSO group was significantly higher compared to the SCD group, while it decreased significantly after dapagliflozin gavage (Figure 3b), indicating that HFD could induce glucose tolerance impairment and dapagliflozin temporarily reduced the blood glucose in HFD-induced obese mice. In addition, dapagliflozin could also slightly reduce the plasma high cholesterol level caused by HFD (Figure 3c). There was no difference in plasma TG levels among the three groups neither (Figure 3d).

### 3.4 Dapagliflozin alleviated hepatic steatosis

**Figure 4.**
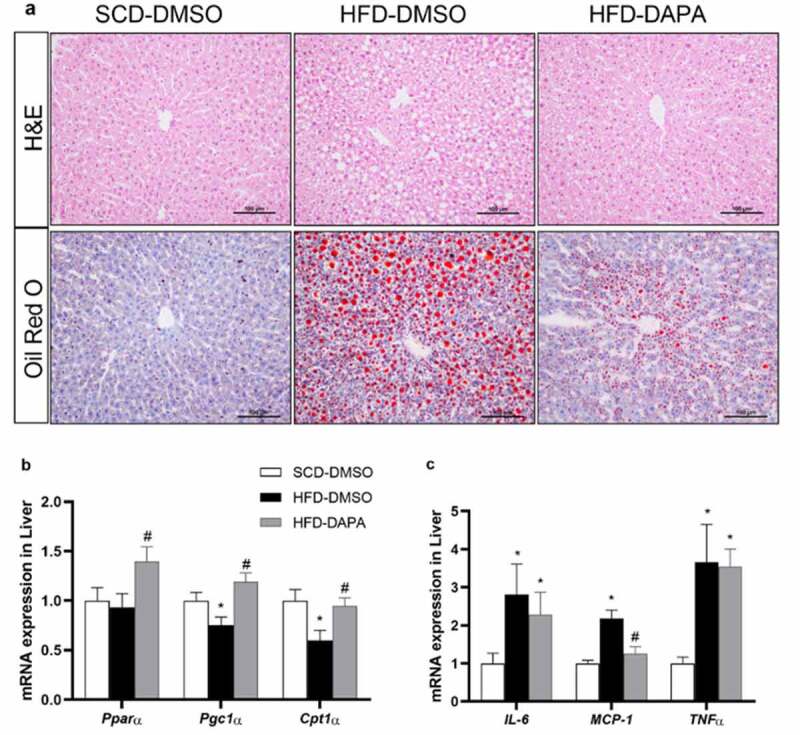
Dapagliflozin alleviated hepatic steatosis. (a) H&E and oil red O stained liver sections. Scale bars = 100 μm. (b) mRNA expression of lipid metabolism-related genes and (d) inflammation-related genes in the liver. Data are presented as means ± SEM, n = 4 per group, 2 duplicated for each sample. *P < 0.05 vs. SCD-DMSO; ^#^*P* < 0.05 vs. HFD-DMSO

Obesity is characterized by excessive fat accumulation in multiple sites of the whole-body, which is often accompanied by a fat liver, leading to hepatic steatosis and NAFLD [4]. H&E staining showed that a large number of hepatocytes were oedema and ballooning-like around the hepatic lobules, and oil red O staining also confirmed the accumulation of large lipid droplets in the liver in HFD group. However, dapagliflozin treatment for 4 weeks significantly alleviated hepatocyte steatosis and reduced lipid accumulation in the liver (Figure 4a). *PPARα*, *PGC-1α*, and *CpT1α* are closely related to lipid metabolism, mainly involved in fatty acid β oxidation, mitochondrial biogenesis, and lipolysis. qPCR results showed that the mRNA expressions of *PPARα, PGC-1α*, and *CpT1α* in the liver of the HFD group were decreased compared with those of the SCD group, while they significantly increased after dapagliflozin treatment (Figure 4b). The occurrence and progression of NAFLD are closely linked to inflammation. The mRNA expressions of inflammatory genes such as *IL-6, MCP-1,* and *TNFα* in the HFD group were increased than those in the SCD group, while dapagliflozin showed no significant reduction on that compared with the HFD-DMSO group except for *MCP-1* (Figure 4c), suggesting that the improvement of dapagliflozin on hepatic steatosis may not be mostly dependent on the inflammatory pathway.

### 3.5 Dapagliflozin inhibited adiposity by promoting WAT browning and repressing BAT whiteness

**Figure 5.**
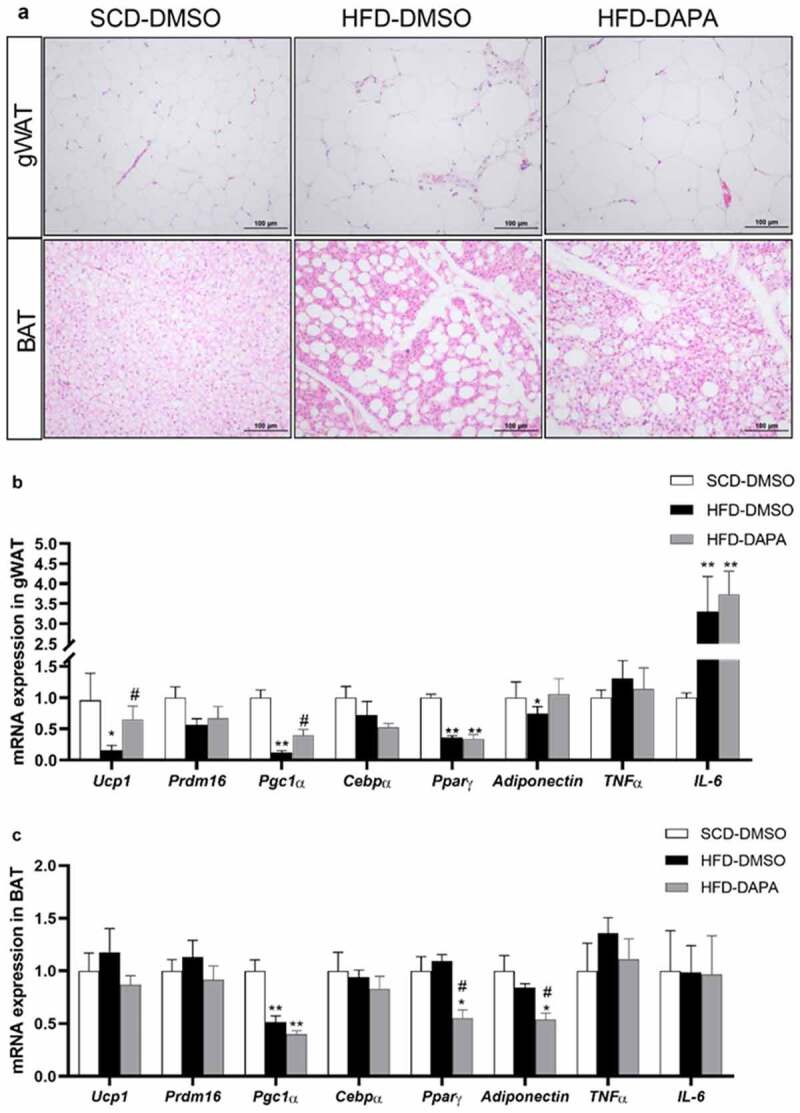
Dapagliflozin inhibited adiposity by promoting WAT browning and repressing BAT whiteness. (a) H&E-stained gWAT and BAT sections. Scale bars = 100 μm. (b) mRNA expression of related genes in gWAT or (c) in BAT. Data are presented as means ± SEM, n = 4 per group, 2 duplicated for each sample. *P < 0.05, ** *P* < 0.01 vs. SCD-DMSO; ^#^*P* < 0.05 vs. HFD-DMSO

Excessive fat accumulation is mainly caused by increases in the number (hyperplasia) or cell size (hypertrophy) of adipocytes [8]. This process is often accompanied by excessive infiltration and retention of pro-inflammatory immune cells, such as macrophages, into adipose tissue, which further aggravates WAT inflammation, leading to insulin resistance and metabolic disorders [5]. H&E staining showed that the HFD could increase the adipocyte sizes in gWAT, the infiltration of mononuclear cells, and also the amounts of white-like adipocytes in BAT. Dapagliflozin could inhibit adipocyte hypertrophy and inflammatory cell infiltration in gWAT and reduce the whiteness of BAT (Figure 5a). qPCR results also confirmed that mRNA expression of browning genes such as *UCP-1* and *PGC-1α* in gWAT decreased significantly after 8 weeks of HFD. Dapagliflozin significantly up-regulated the expression of the above genes, while had no effect on the mRNA expression of adipogenesis genes such as *C/EPBα, PPARγ*, adiponectin, and inflammation genes *TNFα* and *IL-6* (Figure 5b), suggesting that dapagliflozin may be involved in promoting the browning of WAT. In BAT, dapagliflozin had no effect on the mRNA expression of browning or inflammation-related genes, but it could significantly reduce the HFD-induced high expression levels of *PPARγ* and adiponectin (Figure 5c). This was consistent with the observations of H&E staining (Figure 5a), indicating that dapagliflozin could inhibit the white adipocyte genesis in BAT. Thus, dapagliflozin may exert in different ways in WAT and BAT, independent of the inflammatory pathway, synergistically inhibiting the progression of obesity.

### 3.6 Dapagliflozin could improve local oxidative stress in WAT

**Figure 6.**
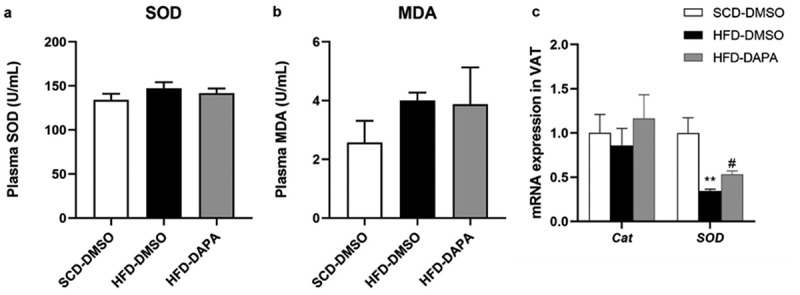
Dapagliflozin could improve local oxidative stress in WAT. (a) Plasma SOD levels. (b) Plasma MDA levels. (c) mRNA expression of ROS related genes in gWAT. Data are presented as means ± SEM, n = 4 per group, 2 duplicated for each sample. *P < 0.05, **P < 0.01 vs. SCD-DMSO; ^#^*P* < 0.05 vs. HFD-DMSO

Oxidative stress is also closely linked to obesity and hepatic steatosis, and thus, we tested the SOD and MDA levels in plasma. Unfortunately, there were no differences among the three groups (Figure 6a,b). Since both of them mainly reflect the systemic ROS level, we further tried to detect the local oxidative stress level in WAT. Although there was no significant change in *Cat* expression among groups, the expression of *SOD* in the HFD group was significantly repressed, and dapagliflozin could reverse the above inhibition to a certain extent (Figure 6c). These results suggest that dapagliflozin may exert its role in improving obesity and hepatic steatosis by affecting the local ROS levels.

## 4 Discussion

The present study provides more evidence on the promising impacts of SGLT2 inhibitors on obesity and its associated metabolic disorders. Several studies have confirmed the protective effects of other SGLT2 inhibitors against obesity in rodents. In a 16-week old HFD pair-fed mice, empagliflozin promoted fat utilization and browning and attenuates inflammation and insulin resistance by polarizing M2 macrophages [25]. Treatment of nondiabetic obese mice with the canagliflozin reduced adiposity and improved glucose tolerance, triggering a fasting-like transcriptional and metabolic paradigm that partly relies on FGF21 un-regulation [26]. Canagliflozin also promoted mitochondrial remodelling in adipocytes through the AMPK-Sirt1-Pgc-1α pathway [27]. Another study reported that luseogliflozin decreased the liver weight and ameliorated steatohepatitis in STZ-treated mice on a HFD without altering the body weight [28]. However, the effects of dapagliflozin on energy homoeostasis and obesity treatment were still not yet proved. In this study, 4-week dapagliflozin administration by gavage in HFD induced obese mice could promote browning of WAT and inhibit adipogenesis in BAT, repressing adipocyte hypertrophy and hepatic steatosis without significant disturbance in weight gain or plasma glycolipid level.

We found that *Scl5a1* expression in WAT of *ob/ob* mice was significantly up-regulated compared with lean mice and was mainly expressed in mature adipocytes, not in SVFs. However, *Scl5a2* expression in adipocytes was close between obese and lean control. Bonner *et al*. also reported similar results in islets from T2DM patients [29]. They found that *Scl5a1* gene expression remained elevated, but *Scl5a2* expression was lower in islets from subjects with T2D, which coincided with an increase in glucagon gene expression [29]. These observations indicated that SGLT1/2 may play different roles in obesity, which still need further exploration.

Previous studies have confirmed that SGLT2 inhibitors have a class effect on weight loss [30,31]. Our present study showed that 4-week dapagliflozin treatment in pre-established HFD induced obese mice reduced the increases of fat mass without significant change in body weight. This inconsistency may be due to the relatively lower dosage used in this study. Liang Xu *et al.* reported SGLT2 inhibited weight gain in a dose-dependent way, finding that only 10 mg/kg dose of empagliflozin, not 3 mg/kg dose, inhibited weight gain in DIO mice [25]. Besides, we failed to detect any difference in blood glucose among groups with or without fasting in DIO mice on chronic dapagliflozin treatment at a dose of 1 mg/kg. This negative result may be due to the deficiency of the HFD model in increasing blood glucose, thus leading to the insignificant glucose-lowering effect of dapagliflozin. However, a single dose of dapagliflozin at the same dose significantly decreased the blood glucose of fasted DIO mice. It was reported that dapagliflozin could trigger glucagon secretion in pancreatic α cells and thus may limit the glucose-lowing effect of fasting [29]. Under feeding state, increased food intake by SGLT2 inhibition might also partly counteract its hypoglycaemic effect [25,32].

Our results confirmed that dapagliflozin could induce the expression of genes (such as *UCP-1* and *PGC-1α*) involved in browning of the adipose tissue in WAT, reverse their down-regulation induced by HFD, and inhibit genes related to adipogenesis in BAT. These observations were similar to the reports on other SGLT2 inhibitors [25,26,32], indicating the class effects of SGLT2 inhibition on adipose tissue and obesity. Dapagliflozin could also decrease lipid accumulation in the liver and alleviate hepatic steatosis. These effects seem to be independent of the improvement of the glycolipid metabolism or tissue inflammation but are related to the alleviation of local oxidative stress in adipose tissue [33]. Several studies reported the risk of SGLT2 inhibitors on liver steatosis by promoting fat lysis and its hepatic transporting [25,34]. However, increasing evidences supported that SGLT2 inhibitors could improve hepatic steatosis in rodents or humans [32,33,35,36]. Our present study indicated that these effects were related to the up-regulation of genes involved in lipid metabolism, such as *PPARα, PGC-1α,* and *CpT1α* in liver, thereby promoting fatty acid β oxidation, mitochondrial biogenesis, and lipolysis.

Our study had some limitations. First, SGLT2 inhibitors have been reported to increase animal’s food intake, and pair-fed control is often recommended. In our study, we did not monitor the food intake of mice during the experiment, which may have some influence on our results. However, we still observed significant changes in fat mass, histology, and certain gene expressions, which are harmonious with others’ reports, making our results credible. Second, there was a lack of deep exploration on the molecular mechanism.

In summary, this study had proved the promising benefits of dapagliflozin on the treatment of obesity and its comorbidities. Dapagliflozin can enhance fat utilization and browning of adipose tissue and improve local oxidative stress, thus inhibiting fat accumulation and hepatic steatosis without disturbance in body weight or plasma glycolipid level. Overall, our study highlights the potential clinical applications of SGLT2 inhibition in the prevention of obesity and related metabolic diseases, such as insulin resistance, NAFLD, and diabetes.

## Supplementary Material

Supplemental Material

## Data Availability

The data that support the findings of this study are available from the corresponding author, C.W., upon reasonable request.
