# Supplementary material for: Sodium glucose cotransporter 2 inhibitor dapagliflozin depressed adiposity and ameliorated hepatic steatosis in high-fat diet induced obese mice
Source: Adipocyte. 2021 Sep 22;10(1):446–55. doi: 10.1080/21623945.2021.1979277 (PMC8475578; doi:10.1080/21623945.2021.1979277)
Supplement: Supplemental Material [file KADI_A_1979277_SM3358.zip › supplementary/Supplementary Table 1.docx]

**Supplementary Table 1. Primer sequences for qPCR**

| **Gene** | **Forward (5’-3’)** | **Reverse (3’-5’)** |
| --- | --- | --- |
| *Scl5a1* | ACGGTATTATCCTGCTGGCG | TCCCCTTGCCACTACAGAGT |
| *Scl5a2* (1^st^) | GCTGCCTATTTCCTGCTGGT | GAACAGAGAGGCTCCAACCG |
| *Scl5a2* (2^nd^) | TGGTGTTGGCTTGTGGTCTA | ATGTTGCTGGCGAACAGAGA |
| *UCP1* | GTGAACCCGACAACTTCCGAA | TGCCAGGCAAGCTGAAACTC |
| *PRDM16* | CCACCAGCGAGGACTTCAC | GGAGGACTCTCGTAGCTCGAA |
| *PGC1α* | ATGTGTCGCCTTCTTGCTCT | ATCTACTGCCTGGGGACCTT |
| *PPARα* | GAGGGTTGAGCTCAGTCAGG | GGTCACCTACGAGTGGCATT |
| *C/EBPα* | CCCTTGCTTTTTGCACCTCC | TGCCCCCATTCTCCATGAAC |
| *PPARγ* | GGAAGACCACTCGCATTCCTT | GTAATCAGCAACCATTGGGTCA |
| *Adiponectin* | AATGGGGCTCCTTCTGGTAAC | GGATGACTCTCCAACGTCCCT |
| *Cpt1α* | AAACCCACCAGGCTACAGTG | TCCTTGTAATGTGCGAGCTG |
| *TNFα* | GATCGGTCCCCAAAGGGATG | CCACTTGGTGGTTTGTGAGTG |
| *MCP1* | CCCTGGGGTTCTTGTGTCTC | CGTTGCTACCTCCACGAAGT |
| *IL-6* | GGCGGATCGGATGTTGTGAT | GGACCCCAGACAATCGGTTG |
| *Cat* | CCAGCGACCAGATGAAGCAG | CCACTCTCTCAGGAATCCGC |
| *SOD* | CAGCATGGGTTCCACGTCCA | CACATTGGCCACACCGTCCT |
| *β-actin* | GTCCACCCCGGGGAAGGTGA | AGGCCTCAGACCTGGGCCATT |
