# Supplementary material for: Sodium glucose cotransporter 2 inhibitor dapagliflozin depressed adiposity and ameliorated hepatic steatosis in high-fat diet induced obese mice
Source: Adipocyte. 2021 Sep 22;10(1):446–55. doi: 10.1080/21623945.2021.1979277 (PMC8475578; doi:10.1080/21623945.2021.1979277)

Sodium glucose cotransporter 2 inhibitor Dapagliflozin depressed adiposity and ameliorated hepatic steatosis in high-fat diet induced obese mice

Supplementary_01: Provided as scatter plot graphs

Tuo Han et al.

Results

Fig 1: *Scl5a2* was overexpressed in *ob/ob* mice, which especially expressed in adipocytes other than SVFs

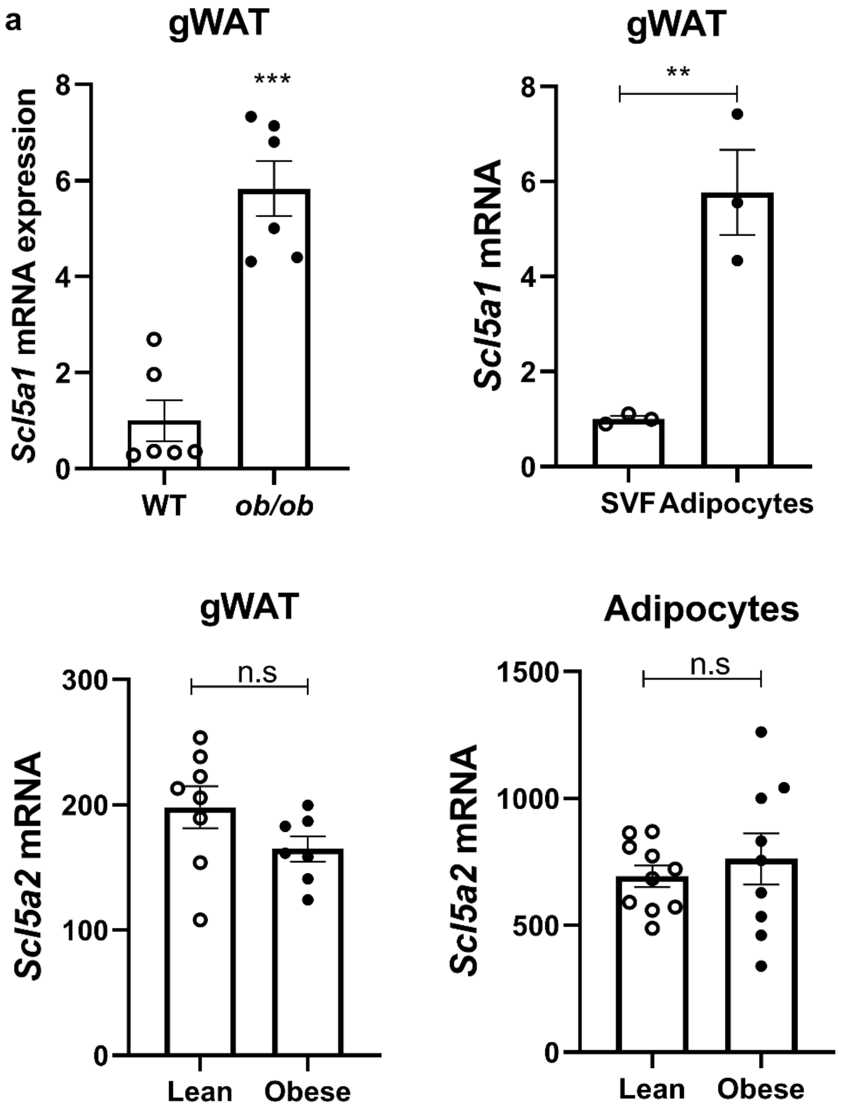

Fig 2: lower dose of DAPA treatment depressed adiposity in HFD induced obese mice

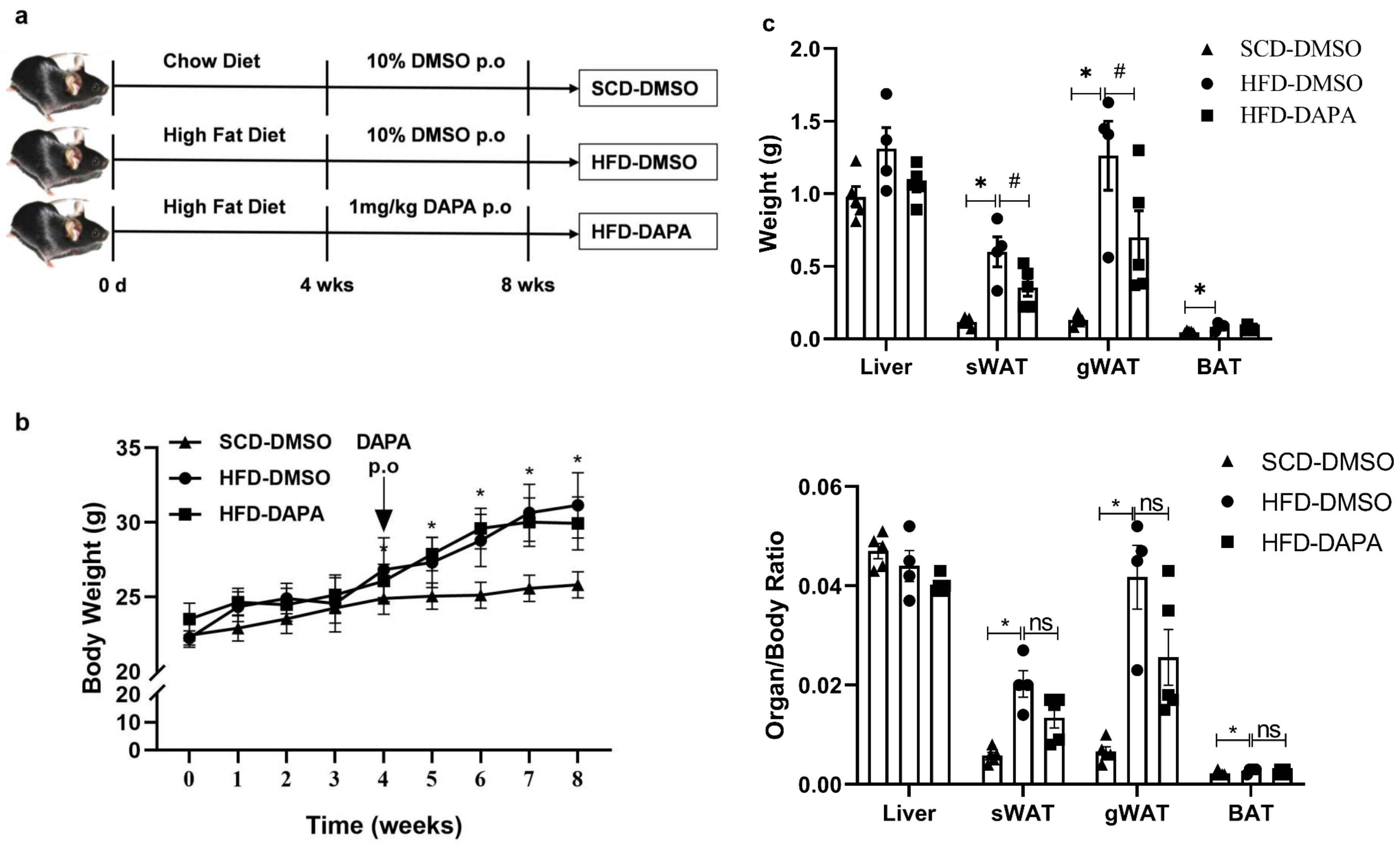

Fig 3: lower dose of DAPA treatment decreased plasma glucose transiently but did not impact plasma lipids in DIO mice

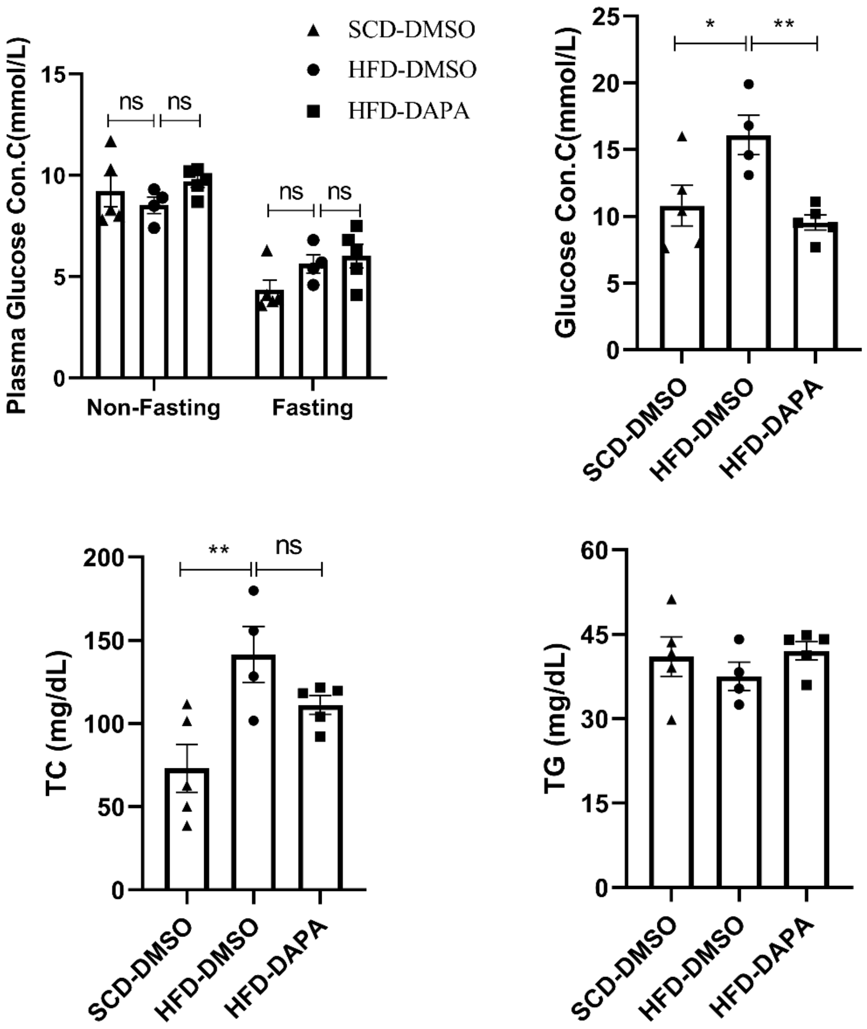

Fig 4: lower dose of DAPA treatment alleviated hepatosteatosis with slightly changes on inflammation

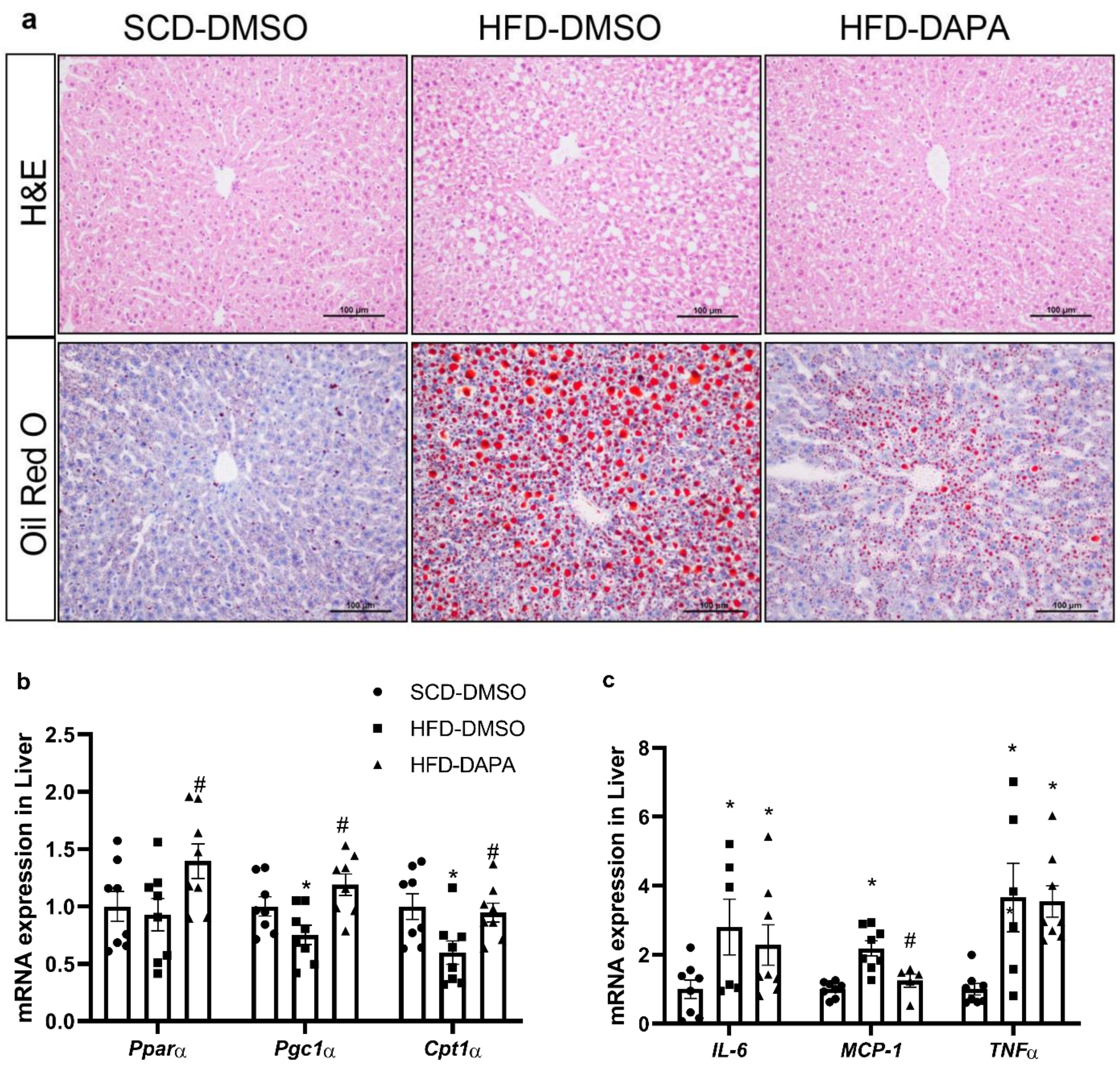

Fig 5: lower dose of DAPA treatment alleviated adiposity by promoting WAT browning and inhibiting BAT whiting

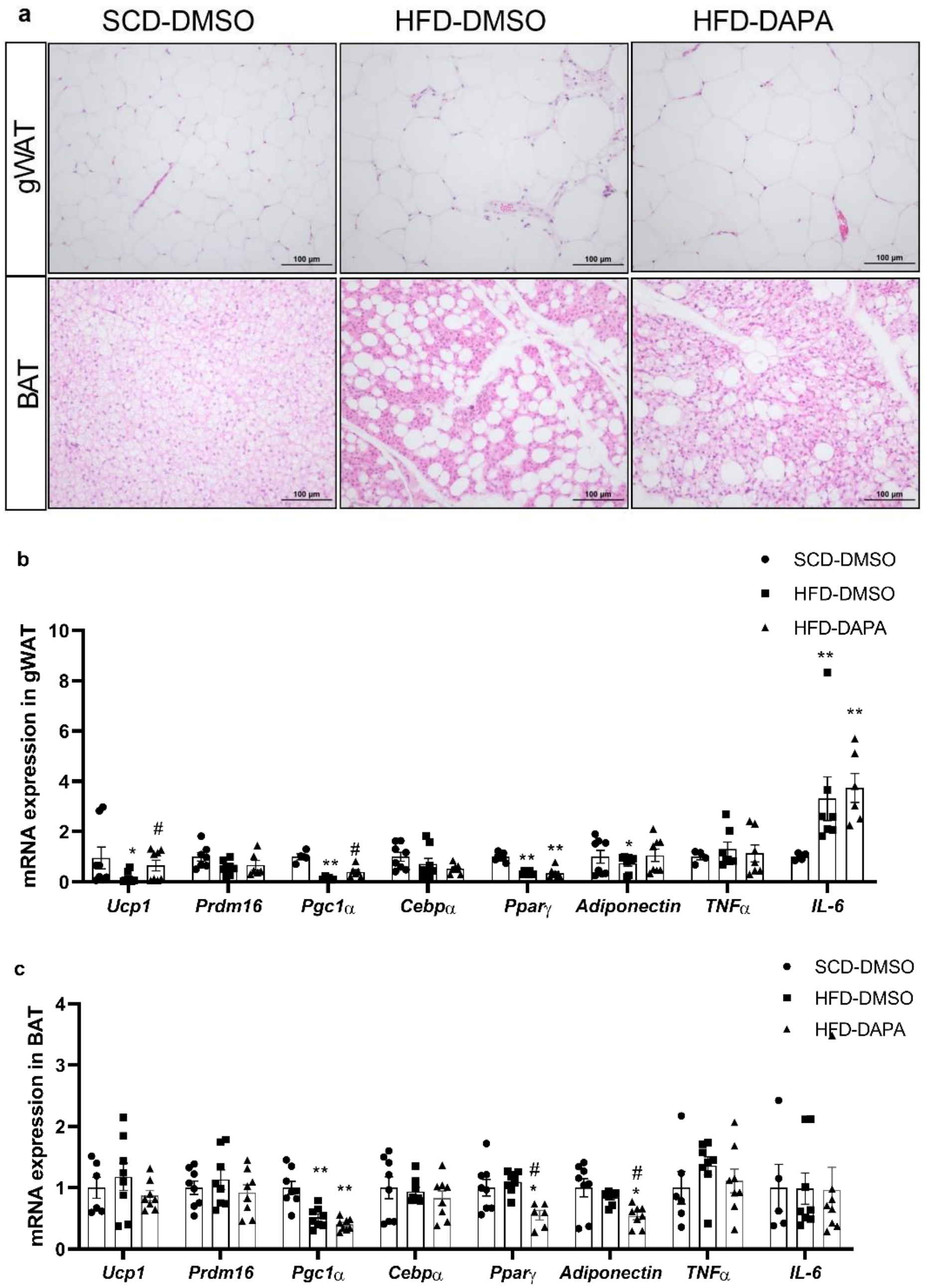

Fig 6: DAPA treatment may increase local capacity of ROS depletion in WAT without affecting systemic ROS

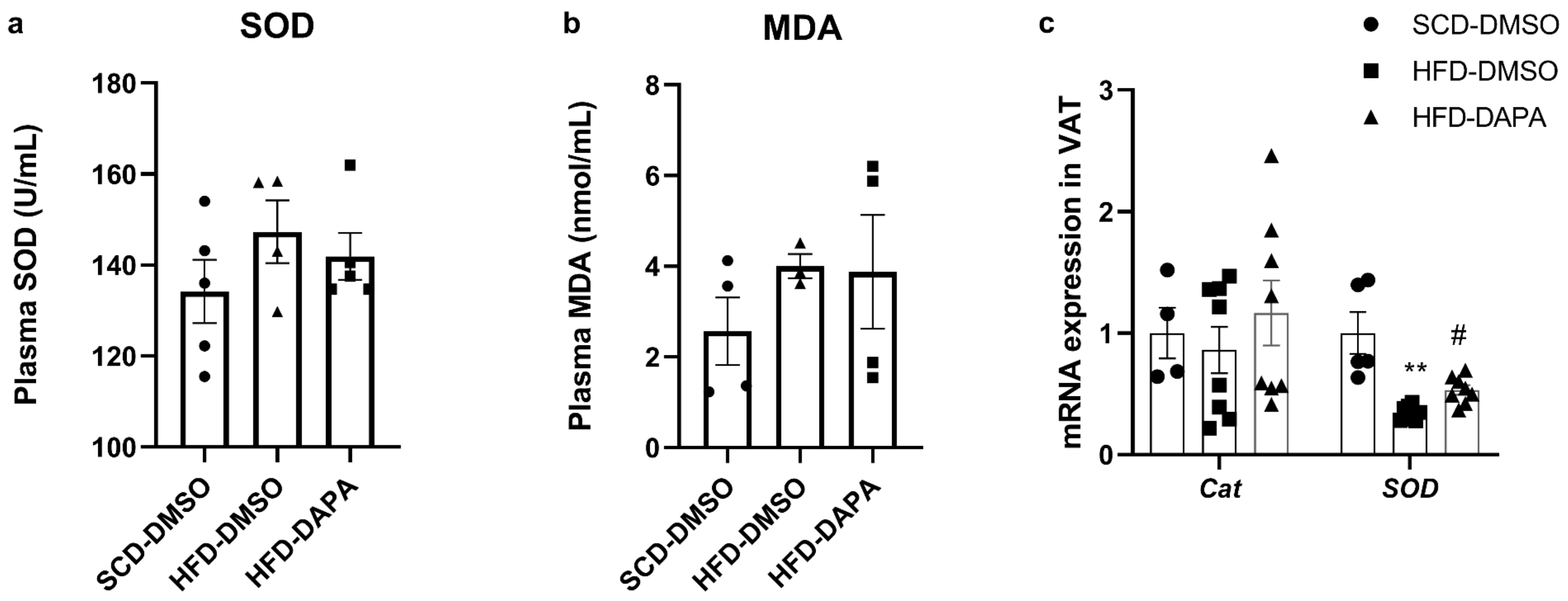

Supplement: Supplemental Material [file KADI_A_1979277_SM3358.zip › supplementary/Supplementary_01 Provided as scatter plot graphs.pdf]
